# A Simple Proteomics-Based Approach to Identification of Immunodominant Antigens from a Complex Pathogen: Application to the CD4 T Cell Response against Human Herpesvirus 6B

**DOI:** 10.1371/journal.pone.0142871

**Published:** 2015-11-23

**Authors:** Aniuska Becerra-Artiles, Omar Dominguez-Amorocho, Lawrence J. Stern, J. Mauricio Calvo-Calle

**Affiliations:** 1 Department of Pathology, University of Massachusetts Medical School, Worcester, MA, United States of America; 2 Department of Biochemistry and Molecular Pharmacology, University of Massachusetts Medical School, Worcester, MA, United States of America; Johns Hopkins University, UNITED STATES

## Abstract

Most of humanity is chronically infected with human herpesvirus 6 (HHV-6), with viral replication controlled at least in part by a poorly characterized CD4 T cell response. Identification of viral epitopes recognized by CD4 T cells is complicated by the large size of the herpesvirus genome and a low frequency of circulating T cells responding to the virus. Here, we present an alternative to classical epitope mapping approaches used to identify major targets of the T cell response to a complex pathogen like HHV-6B. In the approach presented here, extracellular virus preparations or virus-infected cells are fractionated by SDS-PAGE, and eluted fractions are used as source of antigens to study cytokine responses in direct ex vivo T cell activation studies. Fractions inducing significant cytokine responses are analyzed by mass spectrometry to identify viral proteins, and a subset of peptides from these proteins corresponding to predicted HLA-DR binders is tested for IFN-γ production in seropositive donors with diverse HLA haplotypes. Ten HHV-6B viral proteins were identified as immunodominant antigens. The epitope-specific response to HHV-6B virus was complex and variable between individuals. We identified 107 peptides, each recognized by at least one donor, with each donor having a distinctive footprint. Fourteen peptides showed responses in the majority of donors. Responses to these epitopes were validated using in vitro expanded cells and naturally expressed viral proteins. Predicted peptide binding affinities for the eight HLA-DRB1 alleles investigated here correlated only modestly with the observed CD4 T cell responses. Overall, the response to the virus was dominated by peptides from the major capsid protein U57 and major antigenic protein U11, but responses to other proteins including glycoprotein H (U48) and tegument proteins U54 and U14 also were observed. These results provide a means to follow and potentially modulate the CD4 T-cell immune response to HHV-6B.

## Introduction

HHV-6B is a herpesvirus widely spread in human populations. In western countries most individuals get infected with HHV-6B before their second year of life [1] and suffer a relative mild febrile syndrome that recedes spontaneously, and is followed in some cases by a rash referred to as roseola, *exanthema subitum*, or sixth disease [2]. Although most children recover from HHV-6B infection without sequelae, primary infection is a major cause of hospital visits in young children [3]. HHV-6B establishes a lifelong persistent infection that later in life is associated with drug-induced hypersensitivity syndrome with eosinophilia and systemic symptoms (DRESS) [4] and in transplant recipients with graft-versus-host disease (GVHD) [5,6], encephalitis [7–9], and delayed engraftment, among others complications [10]. HHV-6B shares 90% nucleotide sequence identity with HHV-6A [11], but primary infection with this later virus does not seem to produce a clinical disease; later in life, HHV-6A reactivation has been associated with multiple sclerosis (MS) [12].

Adaptive immune responses to herpesviruses are critical in the control of virus replication, as shown by high load of virus in immunosuppressed individuals [13]. Anti-viral drugs, which are not devoid of adverse reactions [14], are used to control viral replication. Immunotherapies have been developed for the human herpesviruses cytomegalovirus (HCMV) and Epstein-Barr virus (EBV) [15]. Similar approaches for HHV-6 are limited by the lack of well-characterized HHV-6 antigens. Recently, some success was achieved in controlling viral reactivation after hematopoietic stem cell therapy using in vitro expanded T cells targeting antigens from adenovirus, BK virus, HCMV, EBV, and HHV-6 [16].

CD4 T cell responses have been shown to be a major determinant in the control of herpesvirus infections in animal models and in humans. In experimentally infected rhesus macaques with simian varicella virus, a model for pediatric varicella zoster virus (VZV), CD4 T cells are more important to control virus than either antibodies or CD8 T cells [17]. Patients with CD4 T cell deficiencies have an increased susceptibility to herpesvirus infections [18]. Transplant patients vaccinated with inactivated varicella vaccine prior to hematopoietic stem cell transplantation have a lower risk of zoster, and protection correlates with the CD4 T cell response to VZV [19]. In immunosuppressed patients, low CD4 T cell counts are correlated with EBV-related diseases [20]; in renal transplant patients, CD4 T cells are critical for recovery of HCMV infection [21] and in HIV patients low numbers of CD4 T cells are correlated with HCMV-associated diseases [22]. In children with primary HCMV infection, virus shedding in urine (a measure of viral load) correlated with delayed CD4 T cell responses [23]. Based on these studies on other herpesviruses, efforts to develop immunotherapy for HHV-6 might benefit from identification of CD4 T cell epitopes.

T cell responses to HHV-6 have been studied mostly in healthy adults [24]. These studies indicate a low frequency of HHV-6-antigen specific T cells in peripheral blood. Challenge of peripheral blood mononuclear cells (PBMCs) with HHV-6 antigens results in the expansion of CD4 T cells that secrete high levels of IFN-γ with Th2-associated cytokines IL-4 and IL-10 also prominent in the CD4 T cell response [25]. A large fraction of these cells have a cytolytic capacity [25–27]. CD8 T cell responses can be shown in ex vivo assays, but only few investigators have been able to established lines or clones [27–30].

A gap in the current understanding of the T cell response to HHV-6 is the definition of immunodominant T cell antigens. The large number of predicted open reading frames (~100) in HHV-6 makes traditional approaches costly and labor intensive. For these reasons, studies to date have limited the number of potential candidates to study. Nastke et al [25] and Martin et al [28] have used computer-based epitope predictions to identify potential epitopes for screening. Gerdemann et al [27], Iampietro et al [29], and Halawi et al [30] used overlapping peptides covering the entire sequence of HHV-6 proteins which are homologues of HCMV immunodominant antigens. Tejada-Simon et al [31] used an even more selective approach, screening for T cell responses from the HHV-6 protein U24 using peptides that share significant homology with self-proteins. Overall, only 16 CD8 epitopes and 14 CD4 T cell epitopes from HHV-6 have been identified (Reviewed in [32]).

In this report, a more comprehensive approach was developed, which combines screening of T cell responses to naturally-produced HHV-6B virus proteins fractionated by gel electrophoresis, mass-spectrometry identification of viral proteins present in positive fractions, and ex vivo testing of peptide pools from these proteins selected based on predicted MHC binding affinity. Using this approach, over 100 new CD4 T cell epitopes from HHV-6B were identified. Robust CD4 T cell responses to a few abundant HHV-6B proteins were observed in all donors, but within these proteins the responses to specific epitopes were quite variable between donors. Fourteen peptides that were recognized by the majority of the donors tested were validated. These peptides might be useful to track and possibly modulate HHV-6B responses in a diverse population.

## Materials and Methods

### Virus

HHV-6B, strain Z29 was kindly provided by S. Jacobson (NINDS, Bethesda, MD, USA). The virus was propagated in SupT1 cells (ATCC # CRL-1942) that were maintained in RPMI 1640 medium (Cellgro, Mediatech Inc., Manassas, VA) supplemented with L-glutamine (2 mM), penicillin (100 U/ml), streptomycin (100 μg/ml) (all from Gibco-Life Technologies, Grand Island, NY) and 10% heat inactivated fetal bovine serum (FBS, HyClone™, GE Healthcare, Pittsburg, PA). Cells were infected by co-culture of non-infected SupT1 and HHV-6B Z29-infected SupT1 at a ratio 5:1. Infected cultures were maintained at a density of 1 x 10^6^ cells/ml in medium supplemented with 5% FBS, and the infection was monitored by measuring the average diameter of the cells and cell viability using a cell counter (Nexcelom Biosciences, Lawrence, MA).

### Antigen preparation

Supernatant from infected cell-cultures was collected when cytopathic effect was evident (i.e., average size of cells was greater than 14.4 μm). Collected supernatant was cleared by sequential centrifugation at 290 x g for 10 min and at 2,000 x g for 20 min. A crude virus pellet was obtained by centrifugation of the supernatant at 39,000 x g for 75 min. The virus pellet was resuspended in 10 mM Tris-HCl pH 7.5 / 150 mM NaCl / 5 mM EDTA (buffer TNE). Infected or non-infected SupT1 cells also were collected. Cells were lysed by repeated freeze/thaw cycles, spun at 1,500 x g for 10 min and the supernatant was collected (cell lysate). HHV-6B crude virus pellet and cell lysates from non-infected and HHV-6B-infected cells were heat-inactivated (at 56°C in a water bath for 1 hour) and used as source of antigens for T cell stimulation.

### Peripheral blood mononuclear cells (PBMCs) and antigen-expanded T cell populations

Blood from healthy donors was collected under a protocol approved by the Medical School Institutional Review Board of the University of Massachusetts (IRB protocol #1668). All subjects provided written informed consent for participation in the study. Donors HLA DRB1 haplotype was determined by the UMass MHC Haplotyping Core Facility. PBMCs were isolated from blood from seropositive donors (Table 1) using Ficoll-Paque (GE Healthcare, Pittsburg, PA) and used immediately in T cell assays. Alternatively, HHV-6B or peptide-expanded oligoclonal T cell lines were generated by a single in vitro expansion with fresh PBMCs, using 5 μg/ml of viral antigens (heat-inactivated HHV-6B cell lysate or crude virus pellet) or 10 μg/ml peptides, in complete RPMI (RPMI 1640 supplemented with 10% AB+ human serum (Interstate Blood Bank, Inc., Memphis, TN), 50 μM beta-mercaptoethanol, 1 mM non-essential amino acids, 1 mM sodium pyruvate, 100 U/ml penicillin and 100 mg/ml streptomycin (all Gibco-Life Technologies, Grand Island, NY). After 3 days, IL-2 (100 U/ml; Chiron, Emoryville, CA) was added to the medium and cells were maintained for 15–21 days and used in T cell assays. A T cell clone recognizing 6BZ_1084 was obtained from peptide-specific oligoclonal T cell populations by limiting dilution. EBV-transformed B cells were produced by infection of PBMCs with EBV as previously reported [33].

**Table 1:** DRB1 haplotype and serologic status of donors used in this study.

Donor Id	DRB1*	HHV-6 IgG^1^	HCMV IgG^2^
037	07:01/07:01	1:1280	-
118	04:04/14:01	1:640	-
130	11:01/13:02	1:320	+
132	13:02/16:01	1:160	-
131	01:01/04:07	1:80	+

1. HHV-6 IgG titer measured by IFA

2. HCMV IgG status measured by ELISA

### Depletion of cell populations

Heparinized whole blood was depleted from CD4, CD8 or CD19 with RosetteSep™ Human Depletion Cocktails following manufacturer’s protocols (StemCell Technologies, Vancouver, Canada).

### HHV-6B and HCMV serologic status

HHV-6B IgG titer was determined in serum using HHV-6 IgG Antibody IFA Kit (Advanced Biotechnologies Inc., Columbia, MD) and HCMV IgG was measured in serum using CMV IgG Enzyme Immunoassay Test Kit (MP Biomedicals, Solon, OH), following manufacturer’s instructions.

### Fractionation of viral antigens

Ten to fifteen micrograms of total protein were loaded in 7.5% SDS-PAGE gel (BioRad Laboratories Inc., Hercules, CA) in duplicate lanes. One lane of the gel was used for coomassie blue staining. A second lane was excised, washed in distilled water for 10 minutes, rinsed, and sliced into ten fractions from top to bottom. Half of each fraction was mashed in a microcentrifuge tube using a pestle micro-homogenizer (Kontes/Kimble Chase, Vineland NJ), and resuspended in 0.5 ml complete RPMI for use in gel band T cell assays. The other half of each fraction was minced with a razor blade and used for protein identification using liquid chromatography and tandem mass spectrometry.

### Gel band T cell stimulation assay

Freshly isolated PBMCs (1x10^6^) were mixed with the gel fraction suspension, and supernatants were assayed for levels of IFN-γ by ELISA (BD OptEIA, BD Biosciences, San Diego, CA) after 120 hours. Alternatively, in vitro-expanded cells (1x10^5^) were incubated with autologous γ-irradiated PBMCs (2x10^5^) and mixed with the gel fraction suspension, and supernatants assayed after 48 hours.

### Liquid chromatography and tandem mass spectrometry (LC-MS/MS)

In-gel digestion protocol and LC-MS^E^ was used to identify proteins in gel fractions. Proteins in the gel pieces were reduced with dithiotreitol, alkylated with iodoacetamide and digested with porcine trypsin (Princeton Separations Inc., Freehold, NJ) (100 ng / mg protein), at 30°C overnight. Peptides were extracted by successive washes with dimethylformamide and 50% acetonitrile / 5% formic acid, dried and resuspended in 5% acetonitrile / 0.1% formic acid. The peptide mix was analyzed using a UPLC–Q-ToF Premier System (Waters Corporation, Milford, MA) operated in MS^E^ acquisition mode. Raw data were processed using ProteinLynx Global Server (PLGS) software v2.3 (Waters Corporation) to generate the precursor and product ions mass list. Each processed file was searched against a database containing HHV-6B Z29, bovine and human proteins sequences (NCBI, download 2009) using the Identity^E^ search algorithm within PLGS.

### Epitope prediction

To predict HLA-DR1 (DRB1*01:01) epitopes we used the HHV-6B Z29 sequence (GI:5733510) from the NCBI database. Each potential 9-mer binding frame was scored using a consensus binding prediction algorithm combining TEPITOPE (P9) [34] and SYFPEITHI [35] scores [36]. Predicted epitopes with scores > -1.0 (P9) and > 18 (SYFPEITHI) were selected for synthesis of peptides in which the 9-mer sequences were extended by the preceding and following four amino acids in the protein sequence. Cysteine residues in the peptides were substituted by serine (in which case, names of peptides were appended with “s”). The NetMHCIIPan 3.0 server [37] (http://www.cbs.dtu.dk/services/NetMHCIIpan/) was used to predict HLA-DR binding affinities (IC_50_ and percentile rank), using the “peptide” mode which reports value for the highest-score binding register in each peptide. For comparison with T cell epitope data in each donor, the HLA-DR allele with higher predicted binding affinity to each peptide was used.

### Epitope screening and validation

IFN-γ ELISpot (eBiosciences, San Diego, CA) assay was performed for the analysis of T cell responses to peptides pools and to individual peptides. Peptides were obtained from Sigma PepScreen (The Woodlands, TX) or 21^st^ Century Biochemical (Marlborough, MA). For screening, freshly isolated PBMCs (3-4x10^5^) were incubated with antigen for 48 hours. For validation, in vitro-expanded cells (2-5x10^4^) plus autologous γ-irradiated PBMCs (5-10x10^4^) were incubated with antigen for 24 hours. Antigen sources included pools of 7–12 peptides (5 μg/ml each), individual peptides, (5 μg/ml), or controls. Positive controls included crude virus pellet purified from culture supernatant, lysate of HHV-6B-infected cells, or a commercial HCMV lysate (Advanced Biotechnologies Inc., Columbia, MD). Negative controls included complete RPMI + DMSO and lysate of uninfected cells. Two wells for each tested pool or peptide, 2–4 wells for positive controls and 4–6 wells for negative controls were usually tested. Secreted IFN-γ was detected following manufacturer’s protocol. Plates were analyzed using the Immunospot Image Analyzer and ImmunoSpot 3.2 software. Heat-maps were obtained using Gene-E (http://www.broadinstitute.org/cancer/software/GENE-E/index.html).

### Statistical analysis of ELISpot data

Positive responses were identified based on a distribution-free resampling approach and an empirical rule. The distribution-free resampling (DFR2x) algorithm described by Moodie et al [38] is available online at [http://www.scharp.org/zoe/runDFR/]. This method was designed to address the specific characteristics of the ELISpot setup. It is based in repeated permutation of samples and controls. If the null hypothesis (H_0_) is true, the permutation of the data between the antigen and the control should not affect the statistics. H_0_ in DFR2x method is defined as: “the mean of experimental wells is less than or equal than twice the mean of the control wells”. The permutation or bootstrap test is combined with a method that introduces adjustment for multiple comparisons (Westfall-Young method). DFR2x is very sensitive to detect intermediate to high positive responses while maintaining a low false positive rate. The method requires at least 4 wells for control and duplicate wells for the experimental samples. The empirical rule (ER) uses the raw data from each plate and donor (spots-forming units (SFU) per well); positive responses were defined as responses higher than the mean of the negative control plus 3 times the standard deviation of the negative control. Next, the variability was assessed by considering the dispersion index (equal to the variance divided by the mean) for each plate and donor; values with dispersion index beyond the 95^th^ percentile were rejected. Finally, individual responses below 6 spots/well were excluded.

### Peptide binding assay

A fluorescence polarization (FP) assay was used to measure the relative binding affinity of peptides to purified HLA-DR1 (HLA-DRA*01:01; DRB1*01:01) as described [39]. Briefly, 100 nM soluble recombinant HLA-DR1 was incubated with 25 nM influenza hemagglutinin HA_306-318_ peptide (acetyl-PRFVKQNTLRLAT) labeled with Alexa488 tetrafluorophenyl ester (Invitrogen, Eugene, OR), together with unlabeled test peptides, included as five-fold dilution series starting from 100 μM. Competition of each test peptide with probe Alexa488-HA_306-318_ for binding to HLA-DR1 was measured by FP (Victor X Multilabel Plate Reader, PerkinElmer, Waltham, MA) after incubation at 37°C for 72 hours. The percent bound at each concentration of test peptide was determined using known values for free (70 mP) and bound (280 mP) Alexa488-HA_306-318_, and the data was fit to a competition binding equation *y* = 1/(1+[pep]/IC_50_), where [pep] is the concentration of test peptide, *y* is the percent probe peptide bound at that concentration, and IC_50_ is the concentration of test peptide required for 50% inhibition of probe peptide binding.

## Results

### IFN-γ responses of freshly isolated PBMCs to HHV-6B

**Fig 1:**
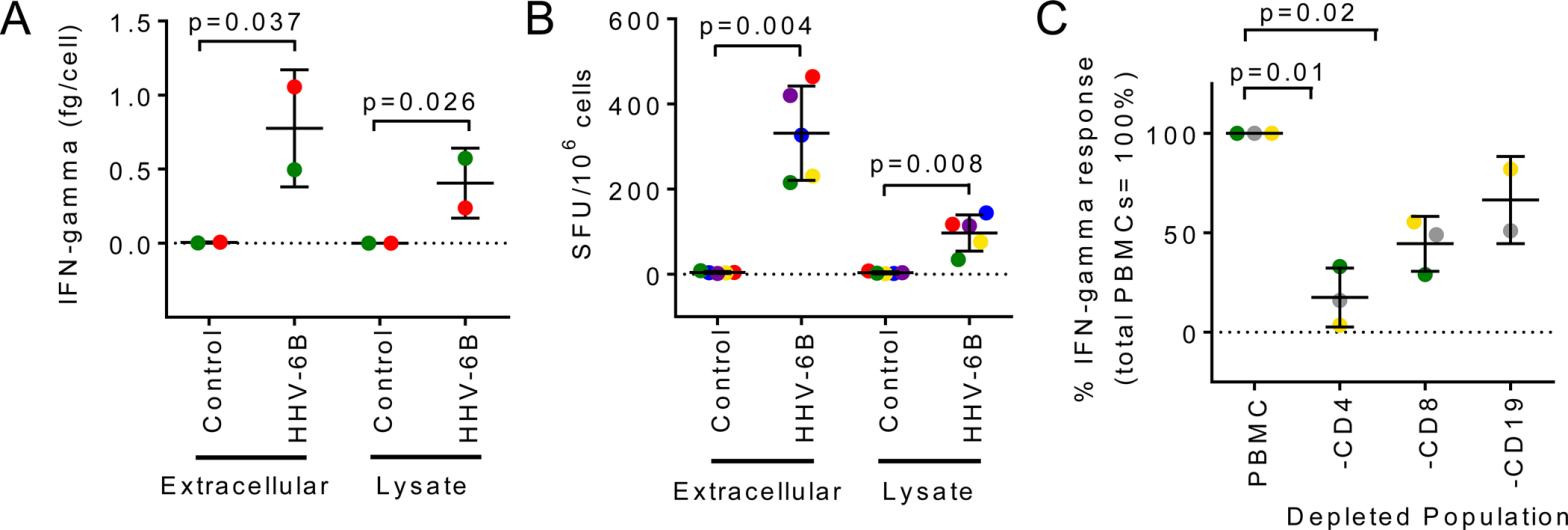
Ex-vivo IFN-γ production by PBMCs from healthy donors in response to HHV-6B antigens (extracellular virus pellet and infected-cell lysate). A. Total IFN-γ in cell culture supernatant measured by ELISA (fg/cell) after 120 hours incubation of PBMCs with antigens. B. Total number of IFN-γ-producing cells measured by ELISpot (SFU/10^6^ cells) after 48 hours incubation of PBMCs with antigens. C. Effect of depletion of CD4, CD8 or CD19 subpopulations on IFN-γ production by total PBMCs, measured by ELISpot (presented as percentage of the response of total PBMCs). Statistical analysis: parametric paired t-test. Each donor is represented by the same colored symbol in the different graphs.

We monitored IFN-γ, a cytokine prominently produced in response to HHV-6 [25,40], in culture supernatants of PBMCs from healthy seropositive donors (Table 1) challenged with two sources of HHV-6B antigens: extracellular virus pelleted from culture supernatant of infected cells and lysate from virus-infected cells. Both sources were heat-inactivated, and relevant negative controls were used. IFN-γ secretion in response to stimulation with both sources of antigens was observed for every donor, while low or non-detectable levels in responses to the negative controls (Fig 1A). The frequency of IFN-γ-producing cells measured using ELISpot assay and reported as SFU per million cells, is shown in Fig 1B for the same set of antigens and controls as in Fig 1A. Low or non-detectable responses were observed with negative controls, while increased responses were detected in the presence of viral antigens, with stronger responses to the extracellular virus. The frequency of IFN-γ-producing cells induced by viral antigens was low, with an average of 300 SFU/10^6^ PBMCs or 0.03 ± 0.01% (n = 5) induced by extracellular virus, and an average of 100 SFU/10^6^ PBMCs or 0.01± 0.01% (n = 5), induced by the infected-cell lysate. We used a depletion approach to study the role of different cell subpopulations in the regulation of IFN-γ production. Depletion of CD4 T cells significantly reduced the levels of IFN-γ as compared to levels obtained with whole PBMCs (about 70% reduction) (Fig 1C), suggesting that CD4 T cells have a major role in the regulation of IFN-γ production by PBMCs. Depletion of other lymphocyte subpopulations had a less marked effect. Altogether, these results indicate that IFN-γ is produced in response to stimulation with HHV-6B viral antigens, and that CD4 T cells are required for efficient production of this cytokine.

**Fig 2:**
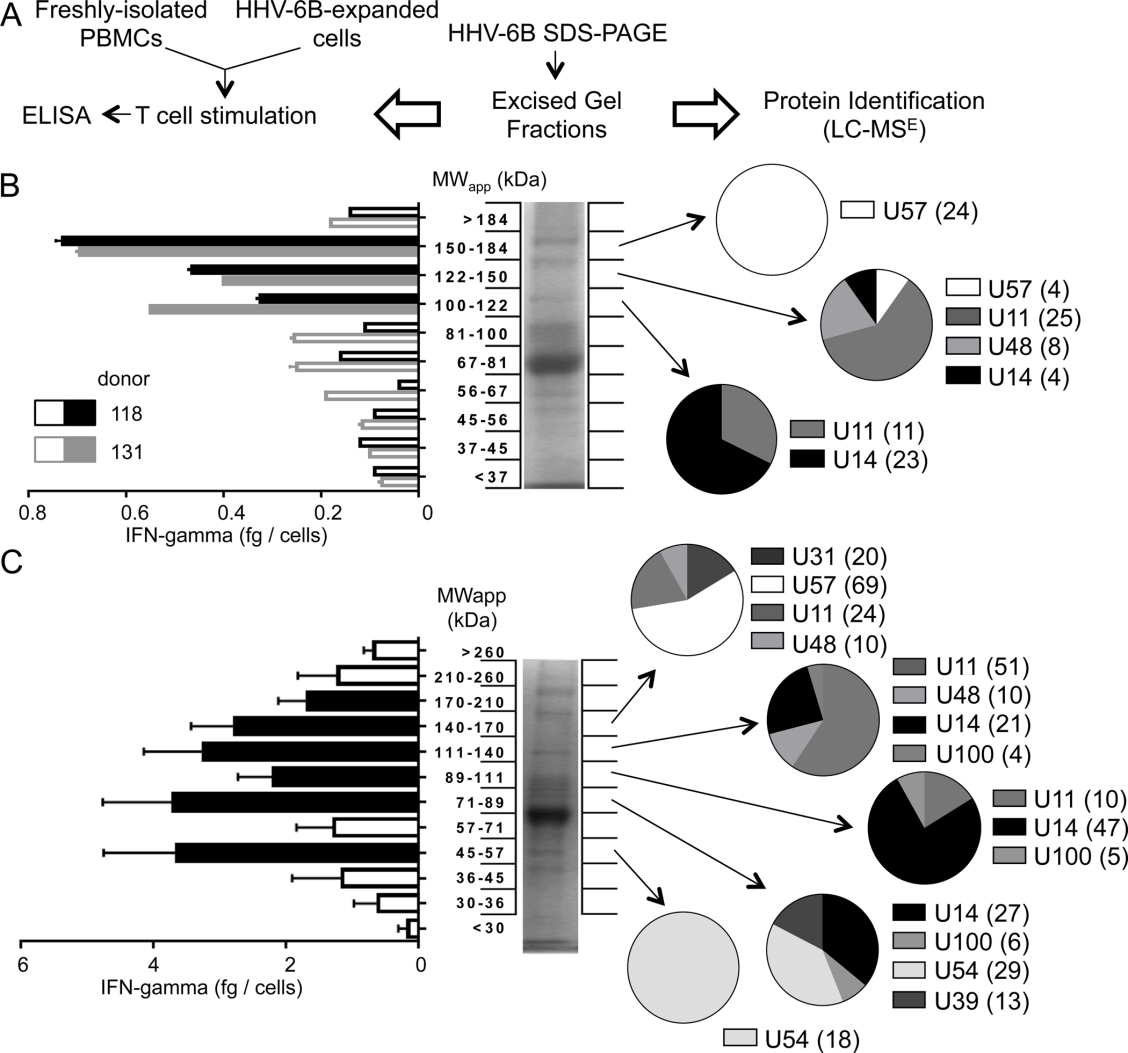
Identification of HHV-6B targets of the IFN-γ response. A. Schematic of the experimental approach: fractionation of proteins by SDS-PAGE; measurement of IFN-γ secretion induced by gel fractions; identification of HHV-6B proteins by proteomics in selected fractions. B and C. IFN-γ production by PBMCs (B) or in vitro HHV-6B-expanded T cell cultures (C), in response to stimulation with gel fractions containing HHV-6B proteins. Middle panels show SDS-PAGE profiles of extracellular virus; left panels show IFN-γ production (fg/cell) by each fraction; right panels show protein identification by proteomics (pie charts show the numbers of tryptic peptides characterized by mass spectrometry used to identify each HHV-6B protein; numbers are shown in parentheses). In B, individual IFN-γ responses of two different donors are shown using black (#118) and grey (#131) bars, with filled bars indicating positive responses (values above the background mean + 3 times standard deviation). Error bars represent standard deviation of replicate measurements for each donor. Filled-bars indicate fractions selected for proteomics analysis. In C, average IFN-γ responses of four expanded T cell cultures (from two different donors); error bars represent the standard deviation. Filled bars indicate positive responses (values above the background mean + 3 times the standard deviation), selected for proteomics analysis.

To identify targets of the IFN-γ response, we combined in vitro cellular immunology and proteomics approaches (Fig 2A). HHV-6B extracellular virus pellet was fractionated using SDS-PAGE, and proteins present in excised gel fractions were used to stimulate freshly isolated PBMCs (Fig 2B) or cells expanded by a single round of in vitro stimulation with HHV-6B (Fig 2C). IFN-γ production in response to excised gel fractions was measured by ELISA and viral proteins present in these fractions were identified by in-gel trypsin digestion and LC-MS^E^. In PBMCs, significant IFN-γ responses to three fractions in the range MW ~100–180 kDa were observed for both donors tested (Fig 2B, filled bars). The fraction at ~150–184 kDa induced the strongest response in both donors. All viral peptides identified in this fraction derive from the HHV-6B U57 protein. For the fraction at ~122–150 kDa, most of the peptides were from U11, but peptides from U48, U14 and U57 also were identified. For the fraction at ~100–122 kDa, peptides from U14 and U11 were identified. Four independent expanded T cell populations derived from PBMCs from 2 donors were tested for IFN-γ production in response to SDS-PAGE fractionated viral antigens. Six gel fractions induced strong IFN-γ responses (Fig 2C, filled bars). A total of 8 viral proteins were identified in these fractions: U57, U31, U48, U11, U14, U100, U54 and U39. An additional experiment was performed using PBMCs, and gel fractions from a cell lysate of HHV-6B-infected cells (data not shown). As a result, 2 additional viral proteins were identified: U41 and U90. A summary of all proteins identified in gel fractions that induced IFN-γ response in PBMCs and/or expanded T cell populations is presented in Table 2.

**Table 2:** Summary of HHV-6B proteins identified by LC/MS^E^ in gel-fractions that induced IFN-γ responses.

Protein Name	ORF	Accession Number	MW (kDa)	Antigen source^1^
Large tegument protein	U31	Q9QJ37	240	Virus
Major capsid protein	U57	Q9QJ26	152	Virus
Major DNA binding protein	U41	P52538	127	Cell lysate
Immediate early protein1	U90	Q77PU6	121	Cell lysate
Major antigenic phosphoprotein pp100	U11	Q69535	96	Virus
Glycoprotein B	U39	P36320	93	Virus, cell lysate
Glycoprotein H	U48	P52543	80	Virus
Phosphoprotein pp85	U14	Q9QJ49	70	Virus, cell lysate
Glycoprotein Q	U100	Q9QJ11	70	Virus, cell lysate
Virion transactivator	U54	Q9QJ29	52	Virus, cell lysate

1. Virus: extracellular HHV-6B virus; Cell lysate: lysate of HHV-6B-infected cells

### Identification of CD4 T cell epitopes in selected HHV-6B proteins

To identify CD4 T cell epitopes within the viral proteins identified by mass spectrometry (Table 2), all potential 9-mer binding frames (core epitopes) in these proteins were evaluated for predicted MHC binding, followed by experimental screening of the T cell responses to the high-scoring subset of the predicted core epitopes. A previously described consensus algorithm was used to predict binding to HLA-DR1 [34–36]. Of the 9,619 total potential 9-mer epitopes present in 10 viral proteins, 463 epitopes predicted to be in the top fifth percentile were selected. Peptides corresponding to these epitopes were synthesized as 17-mers, each composed of the selected 9-mer with 4 flanking residues on each side. A summary of the predictions is presented in Table 3 and peptide sequences, core epitopes, and predicted binding scores are shown in S1 Table. Overall, the synthesized peptides represent 34–65% sequence coverage of the corresponding proteins.

**Table 3:** Summary of DRB1*01:01 binding predictions and IFN-γ responses.

Protein Name	ORF	Len^1^	All predicted^2^	Selected^3^	Coverage^4^	Pools^5^	In 1+ donor^6^	In 4+ donors^7^	Peptides^8^	In 1+ donor^9^	In 3+ donors^10^
Glycoprotein Q	U100	503	608	28	41	3	2	0	-	-	-
Major antigenic phosphoprotein pp100	U11	647	850	36	44	4	4	3	27	21	2
Phosphoprotein pp85	U14	395	602	22	50	3	3	2	15	11	1
Glycoprotein B	U39	830	822	48	45	5	5	1	10	5	1
Glycoprotein H	U48	809	686	45	63	5	5	4	35	25	1
Virion transactivator	U54	413	451	23	65	3	3	2	15	13	2
Major capsid protein	U57	1345	1337	62	44	6	6	3	32	27	6
Large tegument protein	U31	2077	2069	103	52	10	6	1	9	5	1
Major DNA binding protein	U41	1132	1124	64	58	8	5	0	-	-	-
Immediate early protein1	U90	1078	1070	32	34	6	3	0	-	-	-
TOTAL			9619	463	43	53	42	16	143	107	14

1. Protein length (aa)

2. All 9-mers used for predictions

3. Number of selected 9-mers (top 5% of predicted 9-mers); these were synthesized as peptides after extension at the N and C-terminus

4. Protein coverage (%) considering all synthesized peptides

5. Total number of pools per protein. All these pools were tested for T cell responses

6. Number of positive pools in at least one donor

7. Number of positive pools in at least 4 donors

8. Total number of peptides in positive pools (4+ donors); these peptides were tested for T cell responses

9. Number of positive peptides in at least one donor

10. Number of positive peptides in at least 3 donors

**Fig 3:**
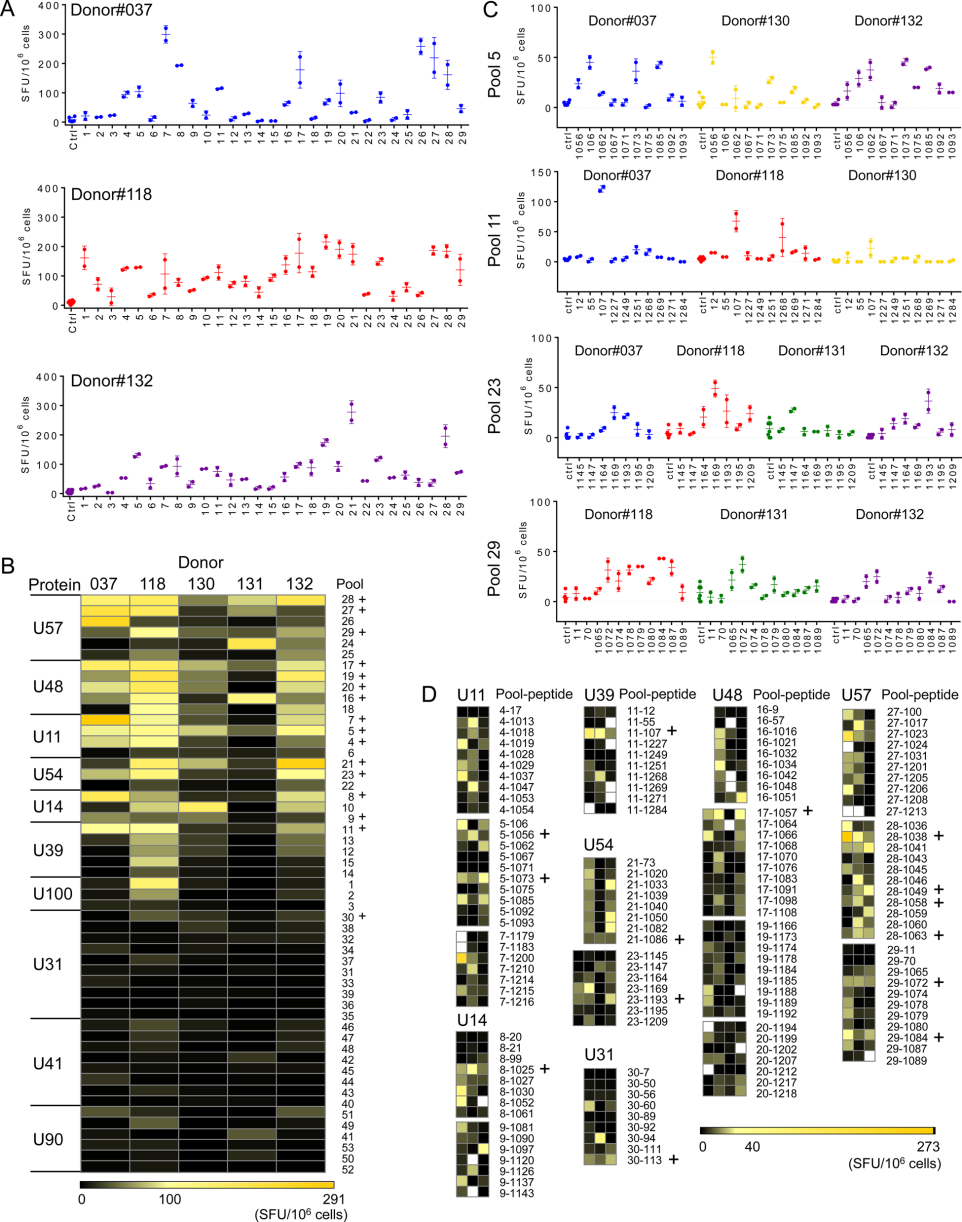
Mapping of the CD4 T cell responses in PBMCs to HHV-6B proteins. A. IFN-γ ELISpot responses (SFU/10^6^ cells) by donors #037, 118 and 132 to pools 1–29. Error bars represent the standard deviation of the replicates. B. Heat maps summarizing the response of all donors (n = 5) to all pools tested (53). Positive responses were assessed by DFR2x and ER analyses (see Methods). Pools for which positive responses were observed by DFR2x in at least 4 donors were selected for further analysis and are indicated by (+). C. IFN-γ ELISpot responses by different donors to individual peptides in pools 5, 11, 23 and 29. D. Heat-maps summarizing the IFN-γ responses to individual peptides present in the pools selected in B. Positive responses to individual peptides were assessed by ER and DFR2x analyses. Fourteen peptides that induced responses in multiple donors were selected for further validation, and are indicated by (+).

To facilitate screening, peptides were grouped by protein into 53 pools of 7–12 peptides per pool (Table 3; S1 Table) according to their predicted binding score, in order to minimize the potential for peptides within a pool to compete for MHC binding. We performed initial screening of IFN-γ responses using PBMCs from 5 normal seropositive donors by ELISpot. Fig 3A shows representative data for three donors and pools # 1 to 29. Fig 3B shows a summary of results from all donors and pools presented as a heat-map, with columns organized by donor and rows by protein and then by pool number. The proteins are ranked vertically by overall T cell response, with peptide pools within each protein ranked according to the average response. The color scale runs from strong responses (up to 291 SFU/10^6^ cells) shown in yellow to no response shown in black. Overall, the magnitude of the T cell responses to the peptide pools was relatively low, with responding T cells frequencies ranging from 0 to ~0.03% for individual donors and peptide pools. Each pool was tested 5 times (265 tests) and overall 133 tests gave a positive response either of two statistical tests, selected to identify relatively sparse responses and taking into account multiple comparisons (see Methods for details). One-hundred and nineteen tests were positive using a distribution-free resampling approach with *p* values less than 0.05 considered positive (DFR2x). One hundred and twelve tests were considered positive using the empirical rule (ER) of mean response minus background greater than three times the standard deviation of the negative control, with rejection criteria for weak and variable responses. A summary of these results is presented in S2 Table. Fifteen pools that elicited positive responses in 4–5 donors by DFR2x were selected for further study; one additional pool (U54-pool 21) was included given the relatively high magnitude of the response and high positive frequency by ER (5 positives out of 5 tests). Selected pools are indicated with a plus sign in Fig 3B.

In order to identify specific epitopes, peptides in each positive pool were screened individually by IFN-γ ELISpot assay. Fig 3C shows representative data for four pools and Fig 3D shows heat-maps summarizing the IFN-γ responses for individual peptides in the 16 pools selected for detailed analysis. One hundred and forty three peptides were tested in donors that showed positive responses for the corresponding pool. The scale ranges from yellow for strong responses (up to 273 SFU/10^6^ cells) to black for no response. Overall, 107 peptides (75% of the peptides tested) induced IFN-γ production in at least one of the donors (S3 Table). As observed for the peptide pools, the magnitudes of the responses to the individual peptides were low, ranging from no response to ~0.03% of PBMC in individual donors. Most of the peptides that induced positive responses (70%) were from proteins U57, U48 and U11 (27, 25 and 21 peptides respectively). Fourteen peptides (Fig 3D, plus sign, and Table 4) showed positive IFN-γ responses by either DFR2x or ER in at least 3 of the five donors. These were selected for validation and additional characterization. The protein U57 was found to be a predominant source of peptides (6 out of 14) showing responses. Epitope information regarding to the peptides studied here will be available in the Immune Epitope Database (http://www.iedb.org/).

**Table 4:** Selected peptides exhibiting IFN-γ response in multiple donors.

Peptide Id	Peptide sequence	Protein	Pool	Response^1^	DRB1* Binding^2^
6BZ_1056	SINKLVYLGKLFVTLNQ	U11	5	26 (4/4)	4600 (225)
6BZ_1073	DVDELKTLYNTFILWLM	U11	5	32 (3/3)	810 (174)
6BZ_1025	LKETILDLAALISNMNL	U14	8	32 (3/3)	2387 (161)
6BZ_107s	LFLAVFLMNSVLMIYSD	U39	11	67 (3/3)	4210 (870)
6BZ_1057	TRTHYLLLAKNGSVFEM	U48	17	33 (4/3)	74 (13)
6BZ_1086s	ELRYFKSGLGISPPSSS	U54	21	19 (3/3)	3858 (482)
6BZ_1193	IVLNIFALPYANVTVSN	U54	23	19 (4/3)	5006 (1537)
6BZ_1038	MGSKIQNLFSAFPIHAF	U57	28	85 (4/3)	324 (39)
6BZ_1049s	HYSNYAIGETIPLQLII	U57	28	21 (4/3)	11 (1)
6BZ_1058	LQLTFFFPLGIYIPTET	U57	28	17 (4/3)	36521 (2817)
6BZ_1063	MQTLLRKSPPQFIVLTM	U57	28	20 (4/3)	7876 (480)
6BZ_1072	NLVSLIRLVKRTISISN	U57	29	26 (3/3)	2276 (95)
6BZ_1084	PIGSIQKLTNILSQYIS	U57	29	21 (5/ 4)	137(5)
6BZ_113	KLFIYIITQAWTASEDS	U31	30	20 (4/3)	37(1)

1. Average IFN-γ response measured in fresh PBMCs; mean value (SFU/10^6^ cells) with the number of donors testing positive over the number of donors tested shown in parenthesis

2. DRB1*01:01 binding reported as IC_50_ in nM, with standard deviation shown in parenthesis

### Validation of selected CD4 T cell candidate epitopes

**Fig 4:**
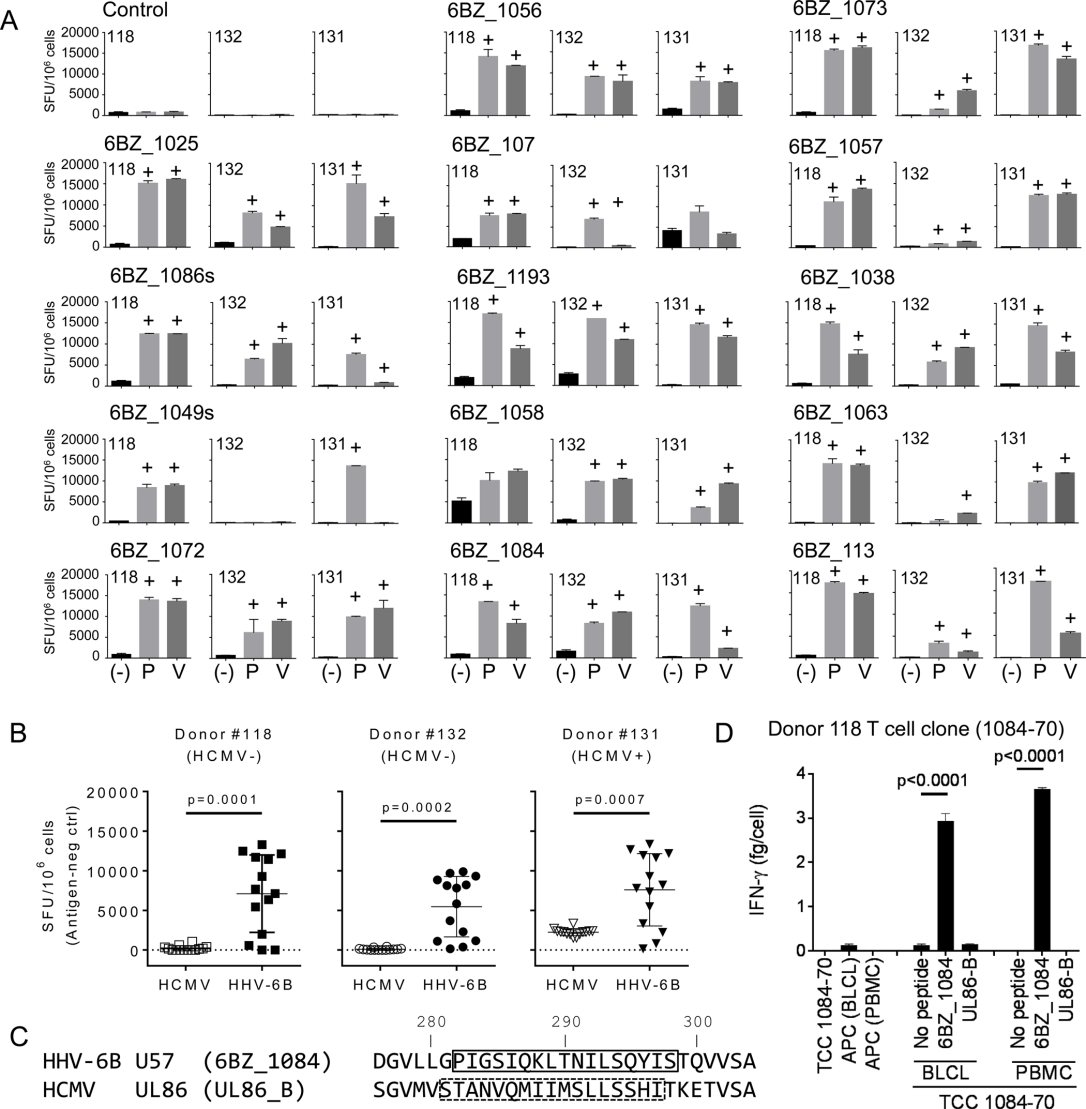
Validation of the IFN-γ response to selected HHV-6B peptides. Peptide-expanded T cell cultures to each of the 14 peptides in Table 4 were generated for 3 donors, and tested for IFN-γ using ELISpot (SFU/10^6^ cells). A. Responses of all expanded T cells to autologous PBMCs pulsed with the peptide (P) or HHV-6B (V). ELISpot negative controls are indicated by (-). Positive responses were assessed by DFR2x and are indicated by (+) on top of the bar. B. Summary of the responses of expanded T cells to autologous PBMCs pulsed with HCMV or HHV-6B for two HCMV seronegative (#118 and 132) and one seropositive (#131) donor (Table 1). P-values for HCMV vs HHV-6B (unpaired t-test) are shown. C. Alignment of homologous HHV-6B U57 and HCMV UL86 sequences in the region of known epitopes (boxes indicate the complete stimulating sequence). D. Response of a 6BZ_1084 CD4 T cell clone to autologous BLCLs or PBMCs pulsed with HHV-6B 6BZ_1084 or with HCMV UL86-B peptides, as measured by IFN-γ ELISA. P-values for no-peptide vs peptide (unpaired t-test) are shown.

One potential complication of peptide-based epitope screening strategies is that cryptic responses possibly might be observed, i.e. reactivity to epitope sequences that are not generated during natural cellular processing of antigens [41]. To evaluate whether any of the fourteen broadly recognized HHV-6B peptides described above represented cryptic epitopes, we evaluated the reactivity of peptide-specific T cell cultures with epitopes generated by natural processing in PBMCs pulsed with HHV-6B antigens. In-vitro expanded cells for each of the fourteen peptides were generated from PBMCs of three different donors. These cells were tested for IFN-γ production in response to autologous APCs pulsed with the exogenous peptide (P) or the virus pellet (V) (Fig 4A). In all cases, the expanded cells that responded to APCs pulsed with the exogenous peptide also responded to APCs pulsed with the virus. These results indicate that epitopes contained in these peptides can be generated from the proteins expressed by the virus via cellular antigen processing mechanisms, and thus represent bona fide viral epitopes.

HHV-6B is related to HCMV, another member of the human beta-herpesvirus family, and it is possible that the T cell specificities that we identified here might cross-react with similar epitopes from HCMV. We tested for this cross-reactivity using the HHV-6B peptide-expanded T cell cultures derived from two HCMV seronegative donors (#118 and #132, Table 1) in an IFN-γ ELISpot assay (Fig 4B). Each culture was tested in parallel with an HCMV antigen preparation and the HHV-6B virus pellet as described previously. No significant reactivity with HCMV was observed for peptide-expanded cultures from the seronegative donors. For an HCMV seropositive donor (#131, Table 1), weak reactivity with the HCMV viral preparation was observed for each peptide-expanded culture (Fig 4B), probably representing HCMV-specific T cells originally present in peripheral blood and persisting in the expanded cultures.

For one of the fourteen HHV-6B epitopes recognized by multiple donors (6BZ_1084), the corresponding sequence in HCMV contains a reported CD4 T cell epitope (Fig 4C) [42]. Although the HHV-6B and HCMV sequences have only 3 identities in the epitope region, at several positions chemically similar residues are present. To test for potential cross-reactivity between the corresponding epitopes from the two viruses, we isolated a T cell clone specific for the 6BZ_1084 epitope from one of the seronegative donors, and tested this for cross-reactivity with the corresponding HCMV epitope, using APCs pulsed with the HHV-6B peptide (6BZ_1084) or the HCMV peptide (UL86-B). No reactivity with the HCMV peptide was observed (Fig 4D), indicating that these epitopes do not cross-react.

### Allele specificity and evaluation of binding prediction algorithms

The peptide-binding prediction algorithm that we used to identify potential HHV-6B epitopes is based on one particular HLA-DR allele, DRB1*01:01. HLA-DR molecules in general have broadly overlapping peptide binding specificities, with HLA-DRB1*01:01 the best characterized of a large group of alleles comprising the main HLA-DR supertype [43]. In previous work we have found predictions based on this allele to be useful in identifying epitopes in donors with a wide variety of HLA-DRB1 haplotypes [25,36]. That was the case in this study as well, with donors of varying haplotypes responding well to the peptides tested, and with the sole HLA-DRB1*01:01 donor not exhibiting stronger or more numerous response than the other donors.

**Fig 5:**
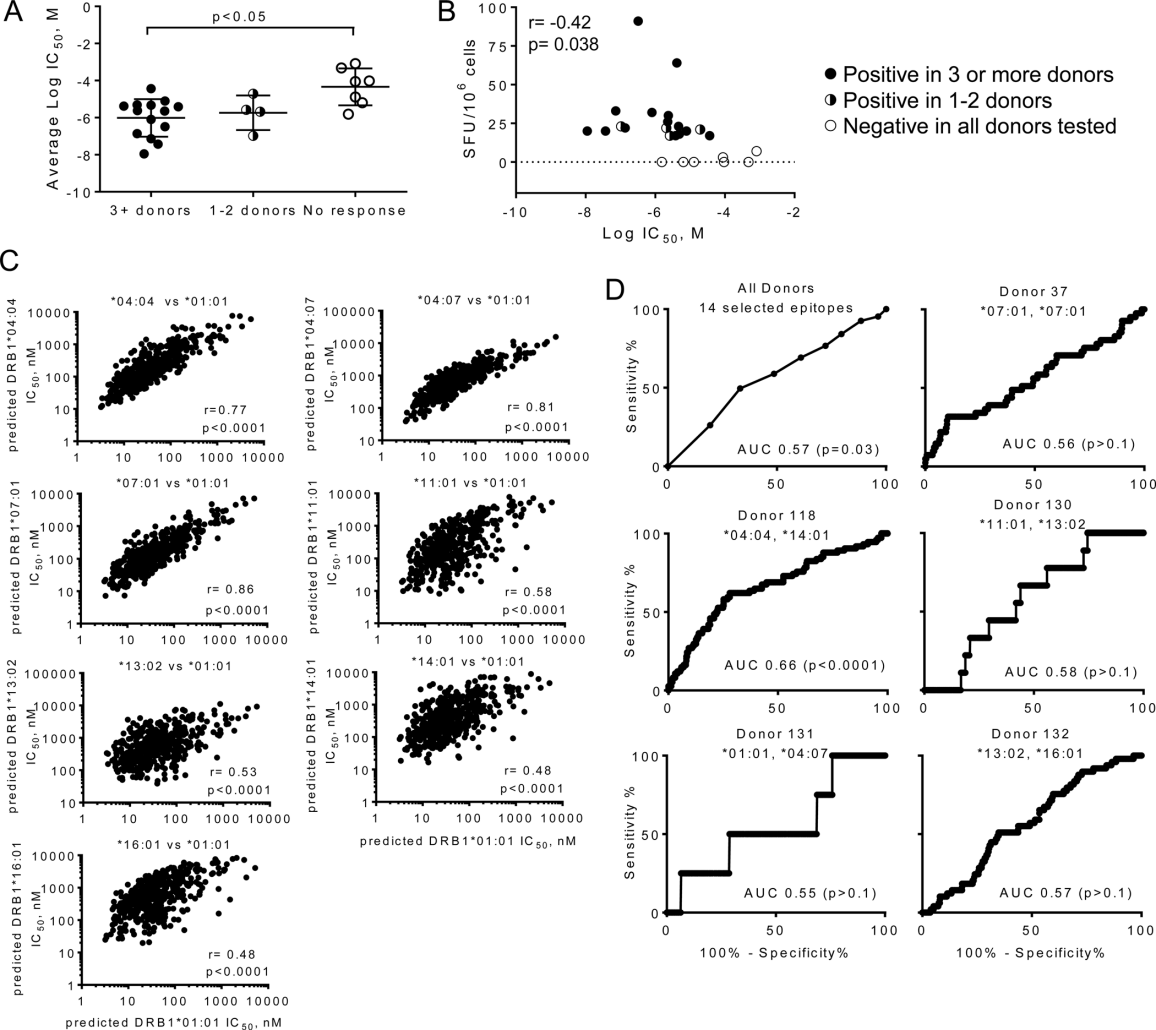
HLA-DRB1*01:01 peptide binding affinity, predicted binding affinity for other HLA-DR alleles, and relation to observed T cell responses. A. Measured HLA-DRB1*01:01 binding affinities grouped according to the frequency of positive responses, for peptides positive in 3 or more donors (n = 14), peptides positive in 1–2 donors (n = 4) or peptides with no positive responses (n = 7). B. Correlation between measured HLA-DRB1*01:01 binding affinity (IC_50_) and magnitude of T cell response (IFN-γ SFU/10^6^ cells); Pearson’s correlation coefficient: -0.42 (p = 0.038). C. Correlation between predicted binding affinities for HLA-DR alleles from donors examined in this study and predicted binding affinity for HLA-DRB1*01:01. D. Receiver-operator characteristic (ROC) analysis relating predicted peptide-binding affinities for all peptides tested in each donor with CD4 T cell responses observed in that donor. *Top left*, ROC analysis for fourteen peptides exhibiting strong responses in multiple donors.

To evaluate the accuracy of the peptide binding predictions, we tested HLA-DRB1*01:01 binding for the 14 peptides exhibiting response in multiple donors, using a fluorescence-polarization competition assay [39] (Table 4, S1 Fig). Each of the peptides bound with measurable affinity, with IC_50_ values ranging from 11 nM to 37 μM. Peptides exhibiting responses in fewer donors had IC_50_ values in the same range, whereas peptides for which T cell response were not observed had significantly lower binding affinity (Fig 5A). In addition to the number of donors responding, the abundance of specific T cells observed in PBMCs from responding donors also correlated with the measured HLA-DRB1*01:01 binding affinity (Fig 5B).

To predict MHC-peptide binding affinity for the other HLA-DR1 alleles present in the donor population, we used NetMHCIIPan 3.0 [37], a pan-specific MHC II neural network-based prediction method (S4 Table). As expected, predicted peptide affinities for the various HLA-DRB1 alleles investigated were highly correlated with that for HLA-DRB1*01:01, with Pearson correlation coefficients varying from 0.48 to 0.81 (Fig 5C). Of the 463 peptides that we had selected using the consensus-based approach to bind to HLA-DRB1*01:01, 442 were predicted by NetMHCIIPan 3.0 to bind to that same protein with IC_50_ < 500 nM. For the other alleles, the number of peptides predicted to have IC_50_ < 500 nM ranged from 166 for DRB1*04:07 to 484 for DRB1*07:01. Most of the donors were heterozygous at the DRB1 locus, such that each donor had at least 321 peptides predicted to bind to one of their HLA-DR types with IC_50_ < 500 nM. NetMHCIIPan 3.0 also provides allele-specific predicted percentile rank values, as the average IC_50_ value is predicted to differ for different HLA-DR proteins. For our donors, the number of peptides predicted to be within the top 10% of all binders varied from 172 to 228 of the total 463 peptides tested, with 23 to 48 predicted to be within the top 1% of all binders (S4 Table). Thus, the set of peptides that we selected using a DR1-based consensus algorithm likely contains many peptides able to bind to other HLA-DR proteins present in our donor population.

We evaluated the ability of NetMHCIIPan 3.0 to predict immunogenicity within this set of peptides on a donor-by-donor basis, using a receiver-operator characteristic (ROC) curve analysis (Fig 5D). The ROC evaluates the tradeoff between the true positive and false positive predictions as the criterion (here predicted IC_50_) is varied. As expected from previous studies of allele-specific epitope prediction for MHC II proteins [44,45] and considering the limited information on allele-specificity in our study, the predictive capacity was relatively low, with areas under the ROC curve (AUC) ranging from 0.56 to 0.66, with only the latter value achieving statistical significance (for donor 118 DRB1*04:04/14:01) (S4 Table). These values can be compared to AUC values of 0.5 for random predictions and 0.8 [44,45] for predictions of well-characterized HLA-DR1-restricted epitopes. For the set of 14 selected epitopes for which we observed responses in multiple donors, and considering responses in all of the donors, the AUC value was 0.57 (p = 0.03). Despite the relatively low predictive value of the NetMHCIIPan 3.0 algorithm for predicting immunogenicity in this study, the algorithm predicted binding affinity quite well. For these peptides for which we had experimental DRB1*01:01 binding data, we compared NetMHCIIPan 3.0 predictions for HLA-DR1 with experimental IC_50_ values. Experimental binding values correlated with predicted IC_50_, with Pearson’s correlation coefficient r = 0.89 and p<0.001 (S2 Fig).

## Discussion

The experimental approach described in this report provides a systematic and cost-effective method to identify immunodominant antigens and uncover T cell epitopes in large-genome pathogens such as HHV-6B. We fractionated an HHV-6B virus preparation using denaturing SDS-PAGE, identified fractions containing T cell antigens by direct analysis of excised gel bands using in vitro cytokine secretion assays, and used mass spectrometry of tryptic digests to characterize viral proteins present in those gel slices showing T cell reactivity. We used a consensus-based epitope prediction algorithm to minimize the number of peptides from the identified viral proteins that needed to be tested, by selecting only candidate epitopes with the best binding probabilities. These peptides were tested in pools and then individually in IFN-γ ELISpot assays. Overall, 107 peptides from ten proteins induced in vitro T responses. Of these, fourteen peptides exhibited strong responses in multiple donors across multiple HLA haplotypes. These fourteen epitopes are generated by natural processing in infected cells, were not cross-reactive with homologs from HCMV, and thus provide a basis for tracking HHV-6B-specific CD4 T cell responses in population and clinical studies.

Gel electrophoresis has been used previously to fractionate antigens for study of T cell responses. A handful of studies in the late 80’s, early 90’s [46–52] and one in 2000 [53] reported cellular responses to individual components in complex preparations of antigens fractionated by SDS-PAGE. These reports were a logical extension of earlier studies using SDS-PAGE-fractionated antigens as immunogens [54–56]. For these in vitro assays, antigens were separated by SDS-PAGE, followed by transfer to nitrocellulose membranes, with release of proteins upon treatment with DMSO [49]. However, inefficient transfer of low and high molecular weight proteins could result in the loss of potential targets [57]. In addition, DMSO used to dissolve membranes could interfere with subsequent cellular assays. Improved methods were developed later, in which samples were fractionated using 2D gel electrophoresis, followed by electroelution of proteins and dialysis; this fractionation method avoided transfer of proteins to membranes and eliminated the SDS in the sample, rendering protein fractions in a suitable solvent for cellular assays [52,53,58]. Bhaskar et al [53] identified eight antigenic fractions of defined molecular weight but did not report identification of the eluted M. tuberculosis antigens. In our protocol, we used a one-dimension fractionation step, sidestepped transfer to nitrocellulose membranes, and identified targets using proteomic workflows involving in-gel tryptic digestion and LC-MS/MS analysis of tryptic peptides. It is possible that improved fractionation methods might allow the identification of additional targets, potentially including low abundance HHV-6B proteins, as in the recent identification of a human tumor antigen [59].

Our method of epitope identification has some advantages relative to other approaches. Screening gel slices for immunogenicity facilitates selection of the major sources of antigens from the mixture, and reduces the number of proteins to be assayed relative to whole-genome or whole-proteome approaches [25,60–62]. In this case, the number of potential candidate proteins was reduced from ~100 HHV6-B proteins (or 36 proteins present in the extracellular virus preparations, unpublished data) to 4 candidates (considering only screening done with PBMCs to characterize high-frequency responses), or 8 candidates (considering screening done with in vitro-expanded cells to include lower-frequency responses). Relative to approaches in which the full set of virus-encoded gene products are screened using either recombinant proteins [60,61,63] or overlapping peptide series [17,62], our method focuses epitope identification work on the subset of proteins that induce strong responses. Relative to approaches in which intact viral preparations are used as a source of antigen, the SDS-PAGE step included in our approach is likely to eliminate viral immune evasion strategies by denaturing all viral proteins. Viral proteins that down-modulate antigen presentation [64–66] and T responses [67–69] are known to limit in vitro T cell responses to many viruses and HHV-6 in particular [64]. In addition, upon electrophoresis, proteins are concentrated in gel regions that upon excision provide enough material to trigger T cell responses. It is also possible that antigens trapped in homogenized gel are more efficiently taken by professional antigen presenting cells than soluble antigens, as shown by experiments with encapsulated antigens [70,71], or that denaturation of viral proteins might facilitate antigen uptake and processing. In most experiments we used extracellular virus preparations as a source of antigens, but we also screened lysates of virus-infected cells in order to account for responses to viral proteins preferentially present inside the infected cell. Two additional proteins (U41 and U90) were identified in this manner, but the responses to peptides pools derived from these proteins were relatively weak and further analysis was not performed.

There are also some caveats to our approach. The primary screening method relies on cellular processing of eluted proteins by APCs present in peripheral blood. The relative efficiency of processing by this route might differ for eluted proteins relative to native proteins present in viral particles or in infected cells, and so the observed immunodominance patterns might not reflect those in vivo. Because of the requirement for cellular uptake of exogenous antigen, responses would be expected to be biased towards CD4 rather than CD8 T cells, although cross-presentation by PBMCs might contribute to observation of CD8 responses. In practice, we observed predominantly CD4 responses. The SDS-PAGE step introduces some limitations: small proteins (<10 kDa) might be lost during electrophoresis, and very large proteins might not elute efficiently. However, we note that we did identify epitopes from several large proteins, including the large tegument protein U31 with >2000 residues. It is possible that limited in-gel proteolytic digestion before elution would improve detection of such large proteins. Finally, the primary screening experiment is constrained by the resolution of SDS-PAGE and the amount of sample available for screening excised gel bands. Reducing the size of the fractions increases the chances that only one viral protein would be present per fraction, but this increases the number of fractions that need to be tested in a single assay, and the availability of cells for the IFN-γ assays is a limiting factor. We used 7.5% SDS-PAGE gels, in order to maximize separation in the 60–150 kDa region, where pilot experiments suggested the majority of the responses where focused, and sliced the gels into ten fractions. Nonetheless, most fractions contained multiple proteins. However, for each of the proteins identified in gel fractions with the exception of U100, multiple epitopes were recognized by our donor population. U100 peptide pools were recognized mainly by one donor and so were not selected for detailed analysis, but strong responses to some individual peptides from U100 were observed in PBMCs from that donor (data not shown).

We used predicted binding to HLA-DR1 (DRB1*01:01) as a criterion to reduce the number of peptides that would need to be tested in the second step of our epitope identification strategy. HLA-DR proteins share a common alpha subunit that contains many of the peptide binding determinants [72], and as result peptide binding specificity is relatively promiscuous, with overlapping peptide binding motifs for different HLA-DR alleles. Among the various alleles for which peptide binding specificity has been characterized in detail, HLA-DR1 can be considered as a “supertype” as its binding motif overlaps with many other alleles [43]. Previous studies have provided experimental support for HLA-DR promiscuity in defined CD4 T cell epitopes [36,73–75]. In the present study, the use of HLA-DR1 binding to sort candidate epitopes seems to be justified, based on retrospective analysis of immunogenicity in all donors of various HLA haplotypes. The experimentally determined HLA-DR1 binding affinities were significantly higher for epitopes recognized by multiple donors as compared to those not recognized by any of the donors tested, and for epitopes associated with stronger responses as measured by IFN-γ as compared to those associated with weaker responses. Moreover, positive responses to selected peptides were observed for each donor tested, regardless of HLA-DR haplotype.

We also retrospectively evaluated the utility of peptide binding predictions available for other HLA-DR alleles in predicting CD4 T cells epitopes from immunogenic HHV-6B proteins. For each of the HLA-DR alleles identified for each donor we used NetMHCIIPan 3.0 [37] to predict binding affinity for the set of 463 peptides tested in this analysis and compared these predictions with the observed T cell responses. For the fourteen strong responses observed in multiple donors, the predicted binding affinity was weakly but significantly associated with presence of T cell responses. However, for most of the individual donors, the predictions were only slightly (and non-significantly) better than random guesses. Even for the HLA-DRB1*01:01-positive donor 131, the predicted IC_50_ values were a poor guide to immunogenicity, despite the high correlation between predicted and observed binding affinity for this allele. Inclusion of HLA-DQ and HLA-DP predictions potentially could increase the sensitivity of this approach. It seems likely that epitope features in addition to MHC II binding affinity play important roles in determining CD4 T cell immunogenicity [76]. Previously, we have reported that resistance to HLA-DM editing is an important factor contributing to antiviral CD4 immunogenicity [77], and it is possible that inclusion of HLA-DM effects could increase the specificity of this epitope prediction approach.

It is noteworthy the diversity of the responses observed. Among 463 peptides tested, 107 provoked an IFN-γ response by circulating CD4 T cells from at least one donor. The profile of the responses was different in the different donors tested. Some proteins have one epitope that appears to dominate the response, for example peptide 6BZ_107 from U39, or peptide 6BZ_113 from U31, while for other proteins such as U48 there is not a clear dominant epitope, and the response observed in pools of peptides seems to be the combination of responses to many peptides. Some HHV-6B proteins induced responses in only specific donors, for example U100 in donor 118, whereas other proteins were able to induce responses in most or all of the donors (U57, U48, U11). The diversity of the response to HHV-6B may have an impact in therapeutic applications. The finding that some of the epitope-specific responses were shared among various donors led us to define a subset of 14 peptides that showed robust responses in the majority of the donors. These could be useful for studying responses to HHV-6B in a heterogeneous population.

The set of epitopes reported here expands on previous studies of the HHV-6B immune response. Of the 107 HHV-6B epitopes reported here, four (6BZ_020, 6BZ_1017, 6BZ_1051, and 6BZ_1066) have been previously observed in studies of the human CD4 T cell responses to HHV-6 (Nastke et al., 2012). Responses to these peptides were not observed in a majority of donors tested here, and so they were not included in the set of 14 commonly recognized peptides. However, the source proteins from which these 14 epitopes were derived overlap with proteins previously identified as sources of CD4 and CD8 epitopes. Five of the seven source proteins (U11, U14, U48, U54, and U57) were previously observed by Naskte et al in studies of the CD4 response [25]. U57 was a prominent source of CD4 epitopes in that study, as in the work described here. Three of these proteins (U11, U14, and U54) also were identified as sources of CD8 antigens [27,28,30].

In summary, we adapted a proteomics-based method for identifying prominent targets of the cellular immune response to the ubiquitous pathogen HHV-6B. The method is based on fractionation and characterization of virus preparations with limited screening of synthetic peptides, and so can be used for pathogens such as HHV-6B, for which recombinant proteins are not available and the large size of genome precludes systematic investigation of all potential epitopes. Using this method we identified over one hundred individual HHV-6B-derived peptides recognized by circulating CD4 T cells in healthy seropositive adults, the vast majority of which represent previously unknown epitopes. Fourteen peptides from seven abundant virion proteins were recognized by robust IFN-γ responses in a majority of donors tested. These represent a set of epitopes that should find application in tracking and potentially modifying cellular immunity to HHV-6B in basic research and clinical studies.

## Supporting Information

S1 Fig Competition assay for binding to DRB1*01:01 by 14 peptides exhibiting IFN-γ response in multiple donors. (PDF)

S2 Fig Correlation between experimental IC_50_ and predicted IC_50_ from NetMHCIIPan 3.0 for 14 peptides exhibiting IFN-γ response in multiple donors. (PDF)

S1 Table Predicted DRB1*01:01 epitopes selected and synthesized peptides. (XLSX)

S2 Table IFN-γ response by peptides pools measured by ELISpot. (XLSX)

S3 Table Peptides for which positive IFN-γ responses were observed by ELISpot. (XLSX)

S4 Table Predicted binding affinity (NetMHCIIPan 3.0) for all peptides and donors DRB1 haplotypes. (XLSX)
