# Supplementary figures and images for: A Simple Proteomics-Based Approach to Identification of Immunodominant Antigens from a Complex Pathogen: Application to the CD4 T Cell Response against Human Herpesvirus 6B
Source: PLoS One. 2015 Nov 23;10(11):e0142871. doi: 10.1371/journal.pone.0142871 (PMC4658110; doi:10.1371/journal.pone.0142871)

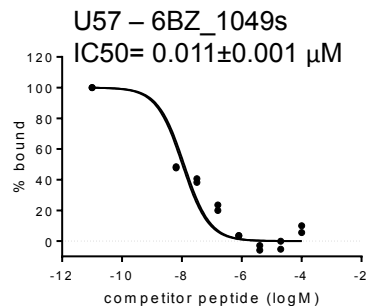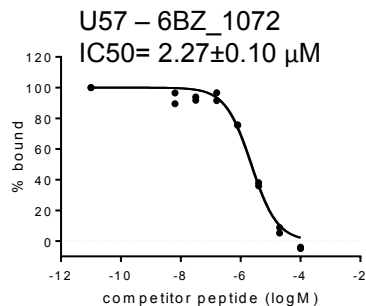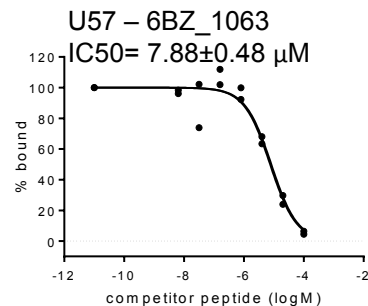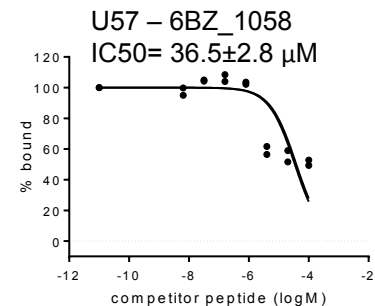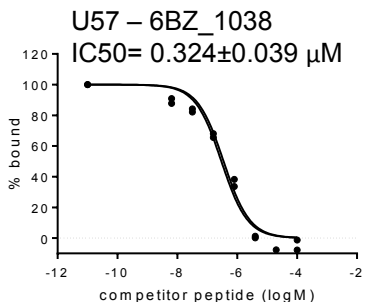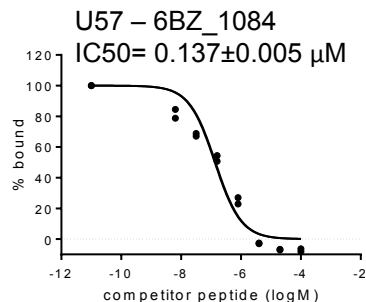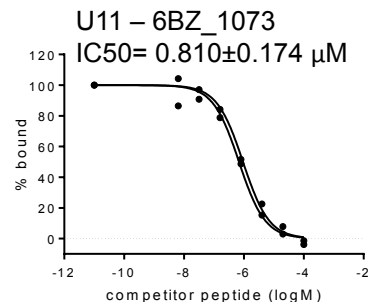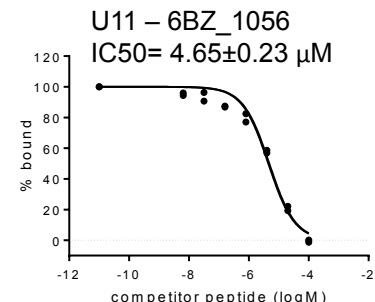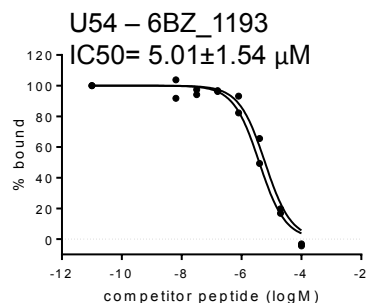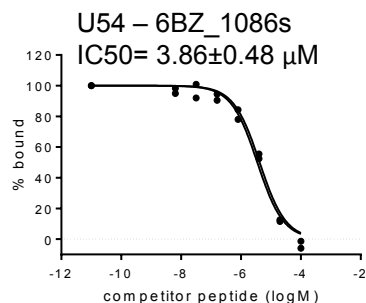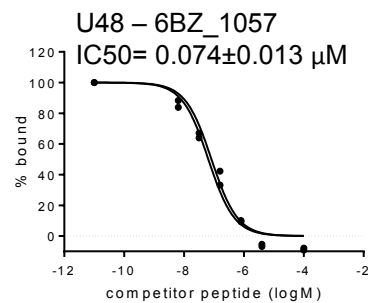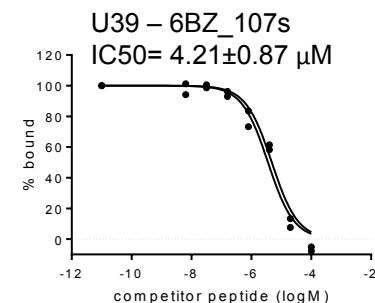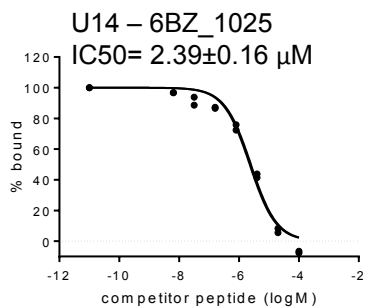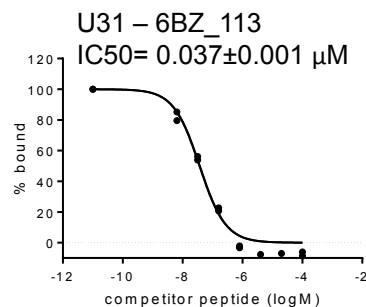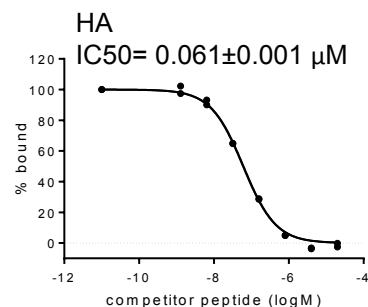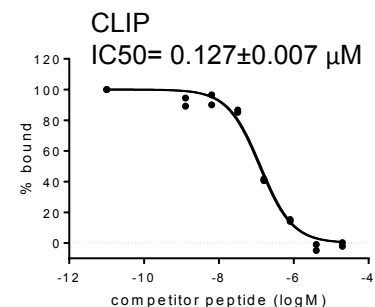

Supplement: S1 Fig — (PDF) [file pone.0142871.s001.pdf]

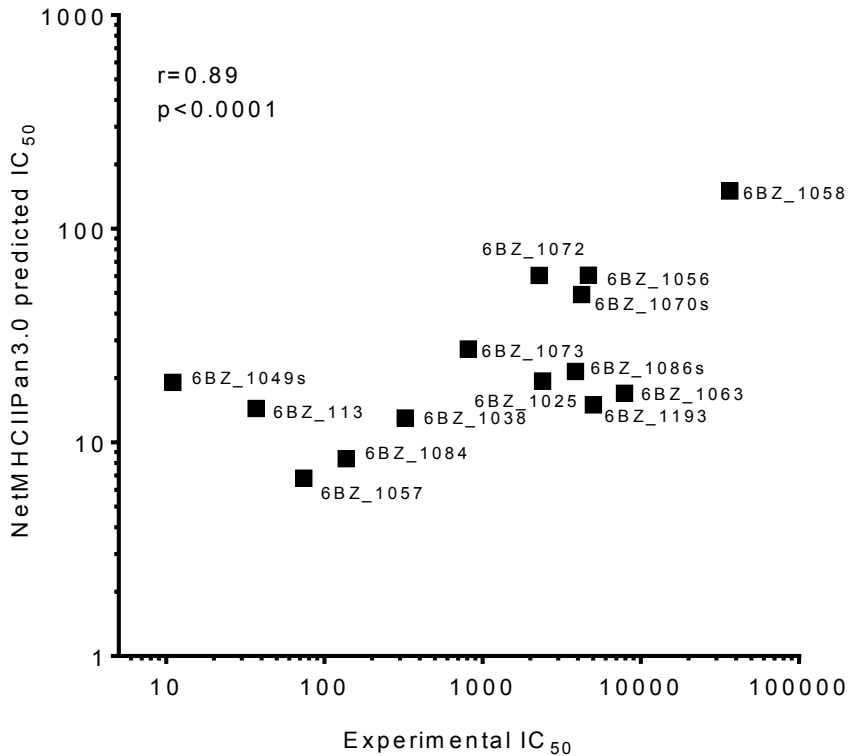

Supplement: S2 Fig — (PDF) [file pone.0142871.s002.pdf]
